# Draft Genome Sequence of Probiotic Lactobacillus brevis TUCO-5E, Isolated from Porcine Milk

**DOI:** 10.1128/MRA.01239-18

**Published:** 2018-11-15

**Authors:** Sandra Rayen Quilodran-Vega, Leonardo Albarracin, Elvira Maria Hebert, Lucila Saavedra, Alexis Fonseca, Alexis Salas-Burgos, Haruki Kitazawa, Julio Villena

**Affiliations:** a Department of Pathology and Preventive Medicine, Faculty of Veterinary Medicine, University of Concepcion, Concepcion, Chile; b Reference Center for Lactobacilli (CERELA-CONICET), Tucuman, Argentina; c Scientific Computing Laboratory, Computer Science Department, Faculty of Exact Sciences and Technology, National University of Tucuman, Tucuman, Argentina; d Department of Pharmacology, University of Concepcion, Concepcion, Chile; e Food and Feed Immunology Group, Laboratory of Animal Products Chemistry, Graduate School of Agricultural Science, Tohoku University, Sendai, Japan; f Livestock Immunology Unit, International Education and Research Center for Food Agricultural Immunology (CFAI), Graduate School of Agricultural Science, Tohoku University, Sendai, Japan; University of Arizona

## Abstract

This report describes the draft genome sequence of Lactobacillus brevis TUCO-5E, a probiotic strain isolated from porcine maternal milk. The reads were generated by a whole-genome sequencing (WGS) strategy on an Illumina MiSeq sequencer and were assembled into contigs with a total estimated size of 2,461,089 bp. A total of 2,455 open reading frames (ORFs) were predicted, including 2,301 protein-coding sequences. The draft genome sequence of L. brevis TUCO-5E will be useful for further studies of specific genetic features and for understanding the mechanisms of its probiotic properties in the porcine host.

## Abstract (precis)

This report describes the draft genome sequence of Lactobacillus brevis TUCO-5E, a probiotic strain isolated from porcine maternal milk. The reads were generated by a whole-genome sequencing (WGS) strategy on an Illumina MiSeq sequencer and were assembled into contigs with a total estimated size of 2,461,089 bp.

## ANNOUNCEMENT

Lactobacillus brevis TUCO-5E (previously L. curvatus TUCO-5E) was isolated in the Bio-Bio region of Chile (1). The TUCO-5E strain was isolated with several other lactic acid bacterial strains from porcine maternal milk and evaluated as a potential probiotic bacterium by studying its antagonistic effects against porcine-associated gastrointestinal pathogens. *In vitro* studies demonstrated that L. brevis TUCO-5E was able to protect intestinal epithelial cells against pathogenic *Salmonella* strains (1). In addition, L. brevis TUCO-5E showed a remarkable ability to protect against Salmonella enterica serovar *Typhimurium* in a mouse model of infection. Adult BALB/c mice preventively treated with the TUCO-5E strain had significantly lower *Salmonella* cell counts in the liver, spleen, and blood than in the controls (1).

A single colony of the TUCO-5E strain was picked for culturing prior to DNA isolation. L. brevis TUCO-5E was cultured for 12 h at 37°C (final log phase) in de Man-Rogosa-Sharpe (MRS) broth (Oxoid, Cambridge, UK), and genomic DNA isolation was performed as described in reference 2. The genomic DNA of L. brevis TUCO-5E was isolated and sequenced with an Illumina MiSeq platform using the 2 × 150-bp paired-end read length sequencing protocol. The generated sequencing reads were filtered to remove low-quality reads and then assembled with SPAdes version 3.11.1 (3). Default parameters were used for bioinformatic analysis. The sequencing protocol generated approximately 81.0× coverage of the genome. The TUCO-5E strain contained 47 contigs of 2,461,089 bp in total. The G+C content was 45.8%. The Rapid Annotations using Subsystems Technology (RAST) server was used for functional annotation of predicted genes (4). A total of 2,455 open reading frames (ORFs) were predicted, including 2,301 protein-coding sequences, 60 tRNAs, 19 rRNAs, and 3 noncoding RNAs (ncRNAs). In addition, 5 clustered regularly interspaced short palindromic repeat (CRISPR) arrays were annotated in the genome and were further confirmed by using CRISPRFinder (5).

RAST analysis revealed that the L. brevis TUCO-5E genome has genes for resistance to tetracycline and vancomycin, which is in agreement with our previous *in vitro* studies (1). The genome was further analyzed with BAGEL4 for the detection of bacteriocins (6), but no bacteriocin genes were detected. One incomplete prophage was detected in the TUCO-5E strain with the use of PHASTER (7).

A gene for bile hydrolysis was found in L. brevis TUCO-5E that could be involved in its ability to survive in the gastrointestinal tract. In addition, clusters of genes involved in the biosynthesis of thiamine, folate, and riboflavin were found in the TUCO-5E genome.

The draft genome sequence of L. brevis TUCO-5E will be useful for further studies of specific genetic features and for understanding the mechanisms of its probiotic properties in the porcine host.

### Data availability.

This whole-genome shotgun project has been deposited in DDBJ/EMBL/GenBank under the accession number QMCB00000000. The version described here is the first version. The SRA/DRA/ERA accession number is ERP106897. The BioSample and BioProject numbers are SAMN09389956 and PRJNA475473, respectively.
